# Compatibility of a New Ocular Surface Dye with Disposable and Bi-Weekly Soft Contact Lenses: An Experimental Study

**DOI:** 10.3390/life14060653

**Published:** 2024-05-21

**Authors:** Mario Troisi, Ciro Caruso, Luca D’Andrea, Michele Rinaldi, Raffaele Piscopo, Salvatore Troisi, Ciro Costagliola

**Affiliations:** 1 Eye Clinic, Department of Neurosciences, Reproductive and Odontostomatological Sciences, University of Naples Federico II, Via Pansini n. 5, 80131 Naples, Italy; troisi165@gmail.com (M.T.); michrinaldi@libero.it (M.R.); raffaele.piscopo2@unina.it (R.P.); ciro.costagliola@unina.it (C.C.); 2 Corneal Transplant Center, Pellegrini Hospital, Via Portamedina alla Pignasecca, 41, 80127 Napoli, Italy; cirocarusoeye@gmail.com; 3 Public Health Department, University of Naples Federico II, Via Pansini n. 5, 80131 Naples, Italy; 4 Ophthalmologic Unit, Salerno Hospital University, 84100 Salerno, Italy; salvatore.troisi@sangiovannieruggi.it

**Keywords:** corneal staining, riboflavin-based dye, contact lenses examination, ocular surface dye, conjunctival staining

## Abstract

Ocular surface staining for assessing corneal and conjunctival epithelium integrity is typically conducted using fluorescein, lissamine green, or rose Bengal dyes. Recently, a novel vital dye, REmark^®^, based on riboflavin, has been proposed for ocular surface examination. In the management of corneal and ocular surface diseases (OSD), the use of contact lenses is integral to therapeutic strategies. This study explores the compatibility of REmark^®^ with four different types of disposable or bi-weekly soft contact lenses. Morphological variations observed under stereomicroscopy and ultraviolet (UV) ray transmittance in the visible spectrum (VIS) were evaluated at 2 and 4 h post-immersion of the contact lenses in both the original fluid and the new dye. The findings indicate no significant differences between the group treated with the original liquid and those immersed in REmark^®^, except for a yellow hue observed in the latter group, which dissipates after 8 h in physiological solution. This study highlights the potential of utilizing the new vital dye for ophthalmologic examinations even in the presence of applied soft contact lenses, offering a promising avenue for improved diagnostic practices and patient comfort.

## 1. Introduction

The evaluation of corneal and conjunctival epithelium typically involves the use of vital dyes such as sodium fluorescein (NaFL), lissamine green (LG), or rose Bengal (RB) staining. NaFL staining aids in identifying corneal cells undergoing apoptosis, primarily highlighting disruptions in intercellular junctions within the corneal tissue [1,2,3,4,5]. While NaFL is highly effective in diagnosing corneal diseases, it lacks utility in detecting conjunctival distress signs, for which RB and LG staining are more suitable [6,7,8,9]. Unfortunately, RB staining poses challenges due to its intrinsic toxicity and photodynamic action [10,11,12]. Additionally, it lacks vital staining properties, as its uptake occurs when the pre-ocular tear film is compromised, staining areas where cultured cells lack coverage by essential components such as albumin and mucin [8,9]. Consequently, many experts advocate for LG over RB in the evaluation of ocular surface disorders, despite its reduced effectiveness in detecting corneal microlesions [13,14,15,16]. To address the limitations of individual dyes, some researchers have proposed a blend of 2% NaFL and 1% LG. This combination offers comprehensive corneal and bulbar conjunctival staining simultaneously, presenting a promising alternative to using single dyes for ocular surface examination and contact lens practice [17]. Common cases requiring ocular surface staining include traumatic lesions, infections, and dry eye conditions [1,18,19]. Soft contact lens wear is a crucial indication for specific staining evaluation, because their prolonged use can cause thinning of the corneal epithelium and stroma, decreased oxygen uptake, increased corneal epithelial microcysts, stromal edema, heightened endothelial polymegathism, conjunctival redness, and limbal neovascularization [14,16,20,21,22,23]. However, in the therapeutic management of corneal and ocular surface diseases (OSD), contact lenses play a crucial role [24]. Hence, the ability to utilize the dye without removing the contact lens would be advantageous. 

Recent studies have demonstrated the efficacy of epithelial staining with a solution of riboflavin sodium phosphate in detecting corneal and conjunctival epithelial defects [25]. These investigations aimed to evaluate the compatibility of the new Riboflavin-based staining technique with the most common disposable or bi-weekly contact lenses available in the market.

## 2. Materials and Methods

### 2.1. Samples Collection and Composition

The study examined four types of soft contact lenses: Purha Daily Water+ by Salmoiraghi & Viganò (EssilorLuxottica Italia S.p.A, Piazzale Luigi Cadorna n. 3, Milan, Italy), Fresh Look One Day Color Contact Lenses by Ciba Vision (Alcon Management S. A. Chemin de Blandonnet 8, Vernier-Geneva, Switzerland), One Day Acuvue by Johnson & Johnson (Johnson & Johnson, New Brunswick, NJ, USA), and Purha Bi-weekly by VistaSì (Vision Group S.p.A., Via Ripamonti 44, Milan, Italy). Each type underwent evaluation with a sample size of 12 contact lenses to ensure test repeatability and obtain average values. Hydrogel contact lenses were chosen over silicone–hydrogel contact lenses due to their superior wettability and capacity to absorb larger quantities of hydrophilic solutions [22], thus facilitating better dispersion of the hydrophilic dye used.

Table 1 summarizes the composition of each type of contact lens and the quantities utilized in the study.

### 2.2. Ocular Surface Dye: REmark^®^

REmark^®^ (SERVImed Industrial S.p.A., Via Tempo del Cielo, 3/5, Roma, Italy) represents a patented ophthalmic solution formulated with riboflavin, available in both multidose and single-dose formats. This solution serves various purposes, including tonometry and ocular surface diagnosis through tear film staining. Riboflavin functions as a stain akin to NaFL. Administered as eye drops, it has exhibited the capacity to yield diagnostic insights comparable to those obtained through double staining with NaFL and LG, all while being better tolerated by patients [25].

Riboflavin solutions have already gained widespread acceptance in ophthalmic applications, facilitating the following:

- Goldmann Applanation Tonometry [26];
- Assessment of ocular surface conditions such as the following:
  ○ Evaluation of contact lenses;
  ○ Detection of abnormal tear production;
  ○ Identification of infections;
  ○ Management of dry eye;
  ○ Measurement of tear break-up time;
  ○ Identification of epithelial abrasions;
  ○ Detection and management of ulcers;
  ○ Evaluation of thin tear meniscus;
  ○ Assessment of corneal integrity loss;
  ○ Management of traumas;
  ○ Evaluation of conjunctival integrity loss;
  ○ Detection of blocked tear ducts;
  ○ Management of corneal edema;
  ○ Identification and removal of foreign bodies.

Utilizing the yellow-green fluorescence properties (up to 565 nm) of its components, diagnostic riboflavin facilitates visualization when illuminated with the cobalt filter typically employed in slit lamps. In comparison to NaFL, diagnostic riboflavin offers several distinct advantages:

- Prolonged Residence: with a residence time on the ocular surface ten times longer than NaFL, diagnostic riboflavin enables the detection of subtle epithelial abnormalities. This extended residence also enhances the analysis of tear film turnover and facilitates the visualization of the black line presence.
- Comprehensive Staining: unlike NaFL, which primarily stains the aqueous part of tears, riboflavin comprehensively stains the entire tear film. This comprehensive staining provides reliable qualitative information through tear break-up time (BUT) analysis.
- Epithelial Stress Detection: diagnostic riboflavin effectively identifies stressed areas in both the cornea and conjunctiva, obviating the need for separate staining with NaFL and LG.
- Compatibility with Contact Lenses: unlike NaFL, diagnostic riboflavin can be used as a diagnostic stain even in the presence of soft contact lenses. The staining effect is temporary, thereby minimizing the risk of adverse effects on the ocular surface.
- Safety Profile: diagnostic riboflavin stains without inducing side effects or damaging the ocular surface [25].

The characteristics of the sample utilized in the study are detailed in Table 2 [27].

### 2.3. Contact Lens Parameters Analyzed

The contact lenses designated for testing underwent prior conditioning in a standard isotonic saline solution. Following this conditioning process, a series of assessments were conducted to ensure a thorough evaluation:

- Visual Inspection: utilizing a stereomicroscope, each contact lens underwent meticulous visual inspection to detect any imperfections, opacity, or irregularities along its edges.
- Dimensional Analysis: employing a profile projector, precise dimensional measurements of each contact lens were taken to assess its overall shape and integrity.
- Transmittance Determination: transmittance measurements were performed across both the UV and visible spectra (ranging from 280 to 800 nm) using a UV–VIS spectrophotometer (CWD 6010, Assiago (MI), Italy). This analysis provided insights into the contact lens’ ability to transmit light across different wavelengths.

Subsequently, the various types of contact lenses were subjected to immersion in the test sample for durations of 2 and 4 h, maintaining a constant temperature of 32.5 ± 2.5 °C throughout the immersion period. Concurrently, contact lenses of the same type were preserved in their original conditioning solution (control) within the designated container under identical conditions. Upon completion of the predetermined immersion period, a comprehensive assessment was repeated, encompassing visual evaluation, dimensional analysis, and transmittance determination across the UV and visible spectra. This post-immersion evaluation aimed to discern any discrepancies between the contact lenses immersed in the test sample and those preserved in their original fluid, facilitating a thorough comparison of their performance and characteristics.

The following instruments and equipment were used:

- Stereomicroscope (Optika, Ponteranica (BG), Italy);
- Profile projector (Nikon, Minato (Tokyo), Japan);
- Spectrophotometer UV–VIS (CWD 6010, Assiago (MI), Italy);
- Incubator (Steinberg systems; Berlin, Germany).

### 2.4. Statistical Analysis

Statistical analysis was conducted utilizing GraphPad Prism version 8 software (GraphPad Software Inc., San Diego, CA, USA). Analysis of variance (ANOVA) followed by Dunnett’s test were employed to assess the significance of differences observed among the tested groups. Statistical significance was determined at a threshold of *p* < 0.05, indicating differences deemed noteworthy. This rigorous analytical approach ensured a robust evaluation and interpretation of the experimental outcomes.

## 3. Results

### 3.1. Visual Inspection 

Moderate yellowing of the contact lenses was noted after immersion in REmark^®^ at both 2 and 4 h (Table 3); the subsequent immersion in physiological NaCl 0.9% solution for 8 h resulted in the complete disappearance of the yellow color. No changes in the contact lenses’ surface were observed by the stereomicroscope.

### 3.2. Contact Lens Diameter

The variations in the diameter of the contact lens were found to be within the limits set by the ISO 18369-2 standard [28], both after immersion in the original solution and in the REmark^®^ for all soft contact lens models tested. In particular, in Purha daily water+ (Salmoiraghi & Viganò, Vigevano, Italy) soft contact lens, the variation of the diameter was 0.050 ± 0.009 mm after 2 h and 0.052 ± 0.008 mm after 4 h of immersion in Remark (ISO 18369-2 limits of 0.20 mm); in Fresh Look One Day Color (Ciba Vision Alcon, Geneva, Switzerland) soft contact lens, −0.017 ± 0.004 mm and −0.024 ± 0.006 mm after 2 and 4 h; in One day Acuvue (Johnson & Johnson, Irvine, CA, USA) contact lens, −0.037 ± 0.008 mm and −0.052 ± 0.011 mm, respectively; and in Purha VistaSì bi-weekly (Salmoiraghi & Viganò, Vigevano, Italy) soft contact lens, −0.051 ± 0.004 mm and −0.002 ± 0.001 mm, respectively (Table 4). 

### 3.3. UV Transmittance

The changes in UV transmittance measured with a UV–VIS spectrophotometer were within the limits of the ISO 18369-2 standard (±5%), both after immersion in the original and in the REmark^®^ solutions for all types of soft contact lens models tested. In Purha daily water+ contact lens, the mean reduction of UV–VIS transmittance was 3.4% ± 0.2% after 2 h and 2.4% ± 0.2% after 4 h; in Fresh Look One Day Color soft contact lens, +0.3% ± 0.04% and −1.24% ± 0.22% after 2 and 4 h; in One day Acuvue contact lens, +0.4% ± 0.06% and −2% ± 0.1%, respectively; and in Purha VistaSì bi-weekly soft contact lens, −0.6% ± 0.07% and +0.5% ± 0.04% after 2 and 4 h, respectively (Table 5).

## 4. Discussion

Analysis of the ocular surface constitutes a crucial aspect in the diagnosis and treatment of various ocular surface disorders. Its effectiveness and cost-efficiency provide valuable insights into the integrity of the ocular surface. It aids in evaluating crucial factors such as the presence of intact tight junctions within the corneal epithelium [2,3], as well as the maturity of the glycocalyx [4]. Typically, the evaluation of both corneal and conjunctival epithelium involves the application of vital dyes such as NaFL, RB, and LG [6]. 

NaFL has stood the test of time as one of the most extensively employed vital dyes, tracing its roots back to the 19th century. Its unique optical properties are noteworthy: it readily absorbs light within the blue spectrum at 490 nm and emits a distinct yellow-green light at a higher wavelength of 530 nm. This characteristic spectral behavior enhances its visibility during staining procedures, especially when complemented with the use of cobalt blue or yellow (blue-free) filters [7]. Despite its widespread use, NaFL exhibits limited penetration into the lipid layer of the corneal epithelium, thus refraining from staining the normal cornea. However, its application unveils valuable insights, particularly when subjected to biomicroscopic observation. This examination often reveals the presence of epithelial cells undergoing apoptosis, offering a glimpse into the dynamic cellular processes occurring on the ocular surface [8]. Furthermore, the efficacy of NaFL staining is accentuated by its propensity for rapid stromal diffusion. Consequently, it serves as a reliable indicator of disruptions in intercellular junctions, primarily within the corneal tissue [2]. While NaFL proves highly effective in diagnosing corneal diseases, its utility in detecting conjunctival distress signs remains somewhat limited, primarily due to inadequate scleral contrast.

For conjunctival staining, RB and LG are heralded as more effective options [8,9]. RB, an anionic water-soluble derivative of fluorescein, was originally believed to exclusively stain devitalized and dead cells, including mucous strands. However, recent evidence suggests that its staining occurs whenever the protection of the pre-ocular tear film is compromised, revealing areas where cultured cells lack coverage by essential components such as albumin and mucin. This newfound understanding underscores the dynamic nature of RB staining, extending its utility beyond initial assumptions. RB’s superiority in the early detection of ocular surface disorders is particularly noteworthy [8,9], providing clinicians with valuable insights into the integrity of the ocular surface. However, despite its diagnostic efficacy, RB’s usage is marred by its intrinsic toxicity and photodynamic action, often resulting in considerable discomfort for patients following instillation. Furthermore, the simultaneous application of lubricants has been shown to interfere with RB uptake, potentially compromising its diagnostic accuracy and effectiveness [9,10,11]. These challenges underscore the need for a careful consideration when employing RB staining in clinical practice, balancing its diagnostic benefits with the associated risks and patient discomfort. RB’s non-vital dye nature contributes to cellular vitality loss post-staining, as evident through instant morphological changes, the subsequent loss of cellular motility, detachment, and eventual cell death. This intrinsic toxic effect is further exacerbated by exposure to light, exacerbating the potential harm it may cause to ocular tissues [11,12]. Given these drawbacks, the clinical utilization of RB is on a downward trend, prompting many experts to advocate for LG as a preferable alternative for evaluating ocular surface disorders. While less irritating and better tolerated, LG falls short in detecting corneal micro-lesions compared to RB [13,14,15]. Despite this limitation, its improved safety profile and the patients’ comfort make it an increasingly attractive option for clinicians seeking reliable diagnostic tools for ocular surface evaluation. 

LG stands out as a synthetically produced organic acid dye featuring two aminophenyl groups. Its application reveals ocular surface epithelial cells lacking protection from mucin or glycocalyx, as well as those that have incurred damage. However, unlike Rose Bengal staining, which is typically recommended in small volumes (25 μL of a 1% solution), standardized application parameters and evaluation methods for LG remain elusive to date [16]. To address the limitations inherent in individual dyes, some authors have proposed a blend of 2% NaFL and 1% LG. This combination offers comprehensive corneal and bulbar conjunctival staining simultaneously, presenting a promising alternative to the use of single dyes for ocular surface examination and contact lens practice [17]. Despite these advancements, further research and standardization efforts are warranted to maximize the diagnostic potential of LG and its synergistic combinations with other vital dyes. The utilization of NaFL and LG dye beyond the limbal area, typically where contact lenses cover, holds significant relevance in investigating symptomatic dry eye patients. Specifically, among soft contact lens wearers experiencing prominent dryness symptoms, elevated LG staining of the bulbar conjunctiva has been observed [29]. 

The limited diffusion of LG in clinical practice has prompted several authors to employ conjunctival staining with NaFL in conjunction with a yellow filter placed in front of the eyepieces. This approach appears to offer heightened sensitivity in detecting conjunctival damage compared to LG alone. By expanding the use of these dyes beyond their traditional applications, clinicians gain valuable insights into the ocular surface health of patients, particularly those experiencing dry eye symptoms exacerbated by soft contact lens wear. This nuanced approach to ocular surface evaluation enables more targeted and effective management strategies for patients with complex dry eye conditions.

NaFL staining with the yellow filter offers the distinct advantage of enabling a simultaneous observation of corneal and conjunctival damage in dry eye patients, eliminating the need for additional vital staining [30].

Ocular surface staining finds indispensable application in various clinical scenarios, including traumatic lesions, infections, and dry eye conditions, highlighting its versatility and significance in diagnosing and managing ocular disorders [1,18,19]. Of particular importance is its role in evaluating patients who wear soft contact lenses. The prolonged use of contact lenses made with hydrogel materials poses significant risks to corneal integrity and functionality. This risk stems from hypoxic stress and mechanical trauma, which can trigger a subclinical inflammatory response. Moreover, in the therapeutic management of corneal and ocular surface disease, contact lenses play a central role. The contact lenses can aid in protecting the ocular surface, promoting healing, and improving patient comfort [24]. Hence, the prospect of utilizing dyes without necessitating contact lens removal could offer significant advantages in these clinical scenarios. 

We have investigated the compatibility of this new riboflavin-based staining technique with the most commonly used disposable or bi-weekly contact lenses available in the market. Such endeavors underscore the ongoing efforts to enhance diagnostic methodologies while ensuring minimal disruption to patient comfort and safety.

The rationale behind selecting these specific types of contact lenses for testing stems from their heightened susceptibility to changes compared to other rigid or semi-rigid contact lenses. These contact lenses are composed of extremely hydrophilic materials, rendering them particularly sensitive to alterations. A hydrated soft contact lens serves as a prime example of a material that harbors bound water, which is trapped within molecular spaces. The polar groups present in polymer molecules exhibit a stronger affinity for water molecules compared to non-polar groups, forming interactions such as ion–dipole, dipole–dipole, or hydrogen bonding. In contrast, the apolar functional groups found in silicone demonstrate a limited ability to bind with water [31,32]. To rigorously evaluate the performance and characteristics of these contact lenses, test conditions were deliberately chosen to be stringent. For instance, the immersion period of 4 h at a temperature of 32.5 °C mirrors conditions akin to prolonged usage scenarios. Specifically, this duration corresponds to four instances of applying eye drops to the same contact lens, with each application lasting approximately 3 min, as estimated for a span of 20 consecutive days.

By subjecting the contact lenses to such rigorous conditions, the study aims to simulate real-world usage scenarios and ascertain their resilience and performance under challenging circumstances. This meticulous approach ensures a comprehensive evaluation and provides valuable insights into the behavior of these contact lenses in practical settings.

At both the 2 h and 4 h marks following the immersion of the contact lenses in the original fluid and the new dye, a comprehensive evaluation of morphological changes under stereomicroscopy and the transmittance of UV rays across the visible spectrum is conducted.

In this analysis, the contact lenses preserved in their original solutions serve as the reference or control samples. The measured values for the examined characteristics of the contact lenses immersed in REmark^®^ are deemed acceptable according to European legislation governing medical devices [27]. Furthermore, any differences observed compared to the control contact lenses treated with the original liquid are deemed nonsignificant. The only notable exception pertains to the yellow appearance exhibited by the contact lenses after immersion in REmark^®^. However, this coloration does not significantly impact UV transmittance and dissipates entirely after an 8 h period in physiological solution.

By adhering to stringent regulatory standards and conducting thorough comparative analyses, the study ensures that the performance and characteristics of contact lenses immersed in REmark^®^ align with established medical device regulations. These findings underscore the safety and efficacy of utilizing REmark^®^ as a diagnostic tool for ocular surface evaluation.

**Figure 1:**
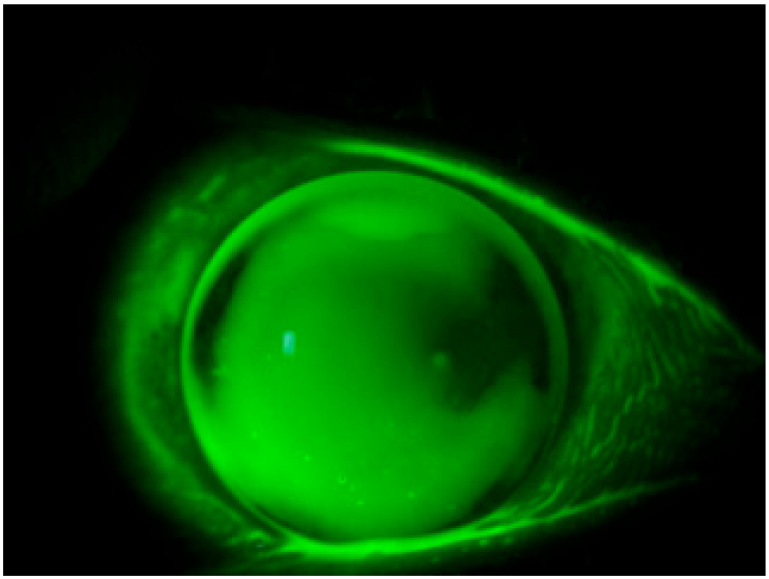
Staining with REmark^®^ while keeping the contact lens worn.

The present study unveils a breakthrough discovery regarding the compatibility of REmark^®^ dye with simultaneous soft hydrogel contact lens wear. This innovative finding demonstrates that soft hydrogel contact lenses efficiently absorb the hydrophilic dye solution, facilitating a comprehensive ocular surface examination without the need for contact lens removal (Figure 1). This novel approach not only overcomes the limitations associated with traditional dyes but also enhances patient comfort and streamlines the diagnostic process.

Hence, this dye holds promise for patients utilizing contact lenses in the therapeutic management of corneal and ocular surface diseases. Soft contact lenses, in particular, are frequently employed for therapeutic purposes, offering a versatile treatment modality in managing various corneal conditions, including bullous keratopathy, corneal erosions, epithelial abnormalities, corneal ulcers, neurotrophic keratitis, neuroparalytic keratitis, chemical burns, and basement membrane disease. It offers structural stability and protection in piggyback lens fitting, as well as post-surgical disorders like post-keratoplasty and post-laser vision correction [24,33,34].

These contact lenses provide therapeutic benefits by creating a protective barrier over the cornea, maintaining corneal hydration, expediting wound healing, and alleviating discomfort associated with these conditions [24,33]. Consequently, the capability to utilize the dye without contact lens removal confers significant therapeutic advantages, both in terms of patient convenience and healthcare resource optimization [24,35,36]. In conclusion, aside from a transient yellow discoloration observed in the second group, which dissipates after 8 h in physiological solution, the data indicate negligible differences between the groups treated with the original liquid and those immersed in REmark^®^. This underscores the potential of REmark^®^ as a valuable tool in the therapeutic management of corneal and ocular surface diseases, offering enhanced convenience and efficiency in patient care.

**Figure 2:**
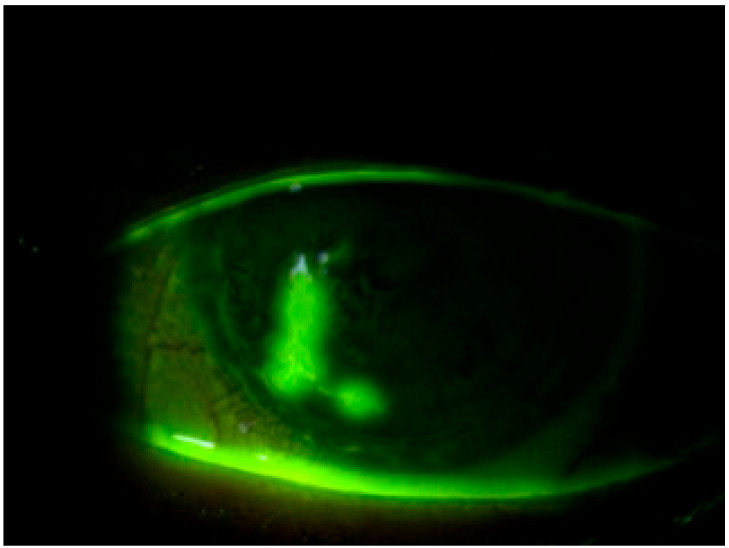
Staining with REmark^®^ in the presence of a therapeutic contact lens for filamentous keratitis.

Moreover, the efficacy of this novel dye in conjunction with a yellow filter for assessing the condition of the bulbar conjunctiva has been investigated, yielding results comparable to those obtained with LG instillation [25,30]. This underscores the versatility and potential of the new dye in comprehensively evaluating changes in both the cornea and conjunctiva, even in the presence of applied soft contact lenses (Figure 2).

## 5. Conclusions

The results derived from the three types of tests, selected based on current reference standards, demonstrate the excellent compatibility of REmark^®^ with the tested contact lenses. These results align entirely with the established reference standards, revealing no indications of soft contact lens deterioration even after prolonged exposure of up to 4 h in the tested dye. Such favorable characteristics permit the utilization of REmark^®^ in conjunction with soft contact lenses, including those with high hydrophilicity, while fully adhering to the requisite safety parameters.

The ability to utilize this dye while wearing contact lenses proves to be exceptionally advantageous, particularly in evaluating the condition of the epithelial surface of the conjunctiva and cornea. This capability holds significant importance for patients afflicted with dry eye and other ocular surface diseases necessitating the use of therapeutic contact lenses due to the presence of corneal epithelial lesions. By facilitating a non-invasive evaluation of ocular surface health, REmark^®^ contributes to enhanced diagnostic precision and streamlined management strategies for these patients, ultimately improving clinical outcomes and patient care. Further studies are needed to compare the results obtained with the other traditional dyes and to evaluate the effect of riboflavin staining on other types of therapeutic contact lenses. 

## Figures and Tables

**Table 1:** Types of soft contact lenses examined and their composition.

Contact Lenses Used for Testing	Composition	Q.ty Tested
Contact lenses type (1): Purha Daily Water+—Salmoiraghi & Viganò	31% Nelfilcon A, 69% Water	12
Contact lenses type (2): Fresh Look—One Day Color Contact Lenses-Ciba Vision	31% Nelfilcon A, 69% Water	12
Contact lenses type (3): One Day Acuvue—Johnson & Johnson	42% Etafilcon A, 58% Water	12
Contact lenses type (4): Purha Bi-weekly—VistaSì	55% Metafilcon A, 45% Water	12

**Table 2:** Detail of characteristics of the dye test (REmark^®^) sample utilized in the study.

Sample Characteristics	
Sample	REmark^®^-Strip from 5 single doses of 0.5 mL
Q.ty tested	50 mL (100 single dose)
LOT	2016/01016.LT
Manufacture date	November 2013
Sterilization method	Filtration

**Table 3:** Contact lens visual variation after immersion in the original liquid and in REmark^®^ at 2 and 4 based on ISO 18369 (Point 9).

Contact Lens Type	Solution Immersed	Hours	Yellowing
Purha daily Water + Salmoiraghi & Viganò	Control	2	No
	REmark^®^	2	Yes
	Control	4	No
	REmark^®^	4	Yes
Fresh Look One Day Color Ciba Vision	Control	2	No
	REmark^®^	2	Yes
	Control	4	No
	REmark^®^	4	Yes
One day Acuvue Johnson & Johnson	Control	2	No
	REmark^®^	2	Yes
	Control	4	No
	REmark^®^	4	Yes
Purha bi-weekly VistaSì	Control	2	No
	REmark^®^	2	Yes
	Control	4	No
	REmark^®^	4	Yes

**Table 4:** Mean change in contact lens diameter after immersion in the original liquid and in REmark^®^.

Contact Lens Type	Solution Immersed	Hours	Mean Change in Contact Lens Diameter (Mean ± SD)
Purha daily Water+ Salmoiraghi & Viganò	Control	2	−0.038 ± 0.007 mm
	REmark^®^	2	−0.050 ± 0.009 mm
	Control	4	−0.005 ± 0.001 mm
	REmark^®^	4	−0.052 ± 0.008 mm
Fresh Look One Day Color Ciba Vision	Control	2	−0.038 ± 0.005 mm
	REmark^®^	2	−0.017 ± 0.004 mm
	Control	4	−0.008 ± 0.002 mm
	REmark^®^	4	−0.024 ± 0.006 mm
One day Acuvue Johnson & Johnson	Control	2	−0.017 ± 0.003 mm
	REmark^®^	2	−0.037 ± 0.008 mm
	Control	4	−0.015 ± 0.003 mm
	REmark^®^	4	−0.052 ± 0.011 mm
Purha bi-weekly VistaSì	Control	2	−0.038 ± 0.006 mm
	REmark^®^	2	−0.051 ± 0.004 mm
	Control	4	−0.050 ± 0.004 mm
	REmark^®^	4	−0.002 ± 0.001 mm

**Table 5:** Mean change in UV transmittance after immersion in the original liquid and in REmark^®^.

Contact Lens Type	Solution Immersed	Hours	Mean Change in UV Transmittance (Mean ± SD)
Purha daily Water+ Salmoiraghi & Viganò	Control	2	−3.8 ± 0.2
	REmark^®^	2	−3.4 ± 0.2
	Control	4	−2.9 ± 0.3
	REmark^®^	4	−2.4 ± 0.2
Fresh Look One Day Color Ciba Vision	Control	2	1.6 ± 0.05
	REmark^®^	2	+0.3 ± 0.04
	Control	4	−3.4 ± 0.27
	REmark^®^	4	−1.24 ± 0.22
One day Acuvue Johnson & Johnson	Control	2	−3.6 ± 0.28
	REmark^®^	2	+0.4 ± 0.06
	Control	4	−0.8 ± 0.11
	REmark^®^	4	−2 ± 0.1
Purha bi-weekly VistaSì	Control	2	−1.0 ± 0.08
	REmark^®^	2	−0.6 ± 0.07
	Control	4	−1.7 ± 0.12
	REmark^®^	4	+0.5 ± 0.04

## Data Availability

Dataset available on request from the authors.
